# Euthanasia Methods in Invertebrates: A Critical Narrative Review of Methodological and Welfare Standards

**DOI:** 10.3390/ani16020222

**Published:** 2026-01-12

**Authors:** Jaco Bakker, Melissa A. de la Garza, Margot Morel, Anne H. Outwater, John Chipangura, Job B. G. Stumpel, Francis Vercammen, Gregory A. Lewbart, Remco A. Nederlof

**Affiliations:** 1 Animal Science Department, Biomedical Primate Research Centre, 2288 GJ Rijswijk, The Netherlands; 2 Michale E. Keeling Center for Comparative Medicine and Research, University of Texas MD Anderson Cancer Center, Bastrop, TX 78602, USA; 3 Broadway Veterinary Group, Unit 1 The Links, Herne CT6 7FE, UK; morelmargot2@hotmail.fr; 4 Department of Community Health Nursing, Muhimbili University of Health and Allied Sciences, Dar es Salaam 65001, Tanzania; 5 Department of Paraclinical Science, University of Pretoria, Onderstepoort 0110, South Africa; 6 WILDLANDS Adventure Zoo Emmen, 7801 BA Emmen, The Netherlands; 7 Centre for Research and Conservation, Royal Zoological Society of Antwerp, K. Astridplein 20-26, B-2018 Antwerp, Belgium; 8 College of Veterinary Medicine, North Carolina State University, Raleigh, NC 27607, USA; 9 Independent Researcher, 2861 XZ Bergambacht, The Netherlands

**Keywords:** euthanasia, invertebrates, welfare, pain, killing, ethics, guidelines, culture of care

## Abstract

Invertebrates are increasingly consumed and kept as pets, research models, and in zoological exhibits, creating a growing need to better understand their clinical management and welfare. However, the knowledge regarding nociception, pain perception, and euthanasia in invertebrates remains limited, and standardized protocols are largely absent. Current guidelines are incomplete, often anecdotal, and omit several major invertebrate phyla. To address this gap, we conducted a narrative review of the literature, aiming to critically evaluate existing euthanasia methods, associated welfare implications, and opportunities for refinement. The amount of peer-reviewed species-specific literature is limited and scattered. In addition, most described methods are insufficiently studied and/or do not align with our definition of euthanasia. Based on the available literature, and to provide practical guidance despite these limitations, we propose a two-step approach to invertebrate euthanasia. The first step consists of inducing anesthesia to achieve loss of responsiveness, followed by a second step; a terminal procedure involving physical or chemical destruction of the brain or major ganglia. Our review focuses on the application of this two-step approach. The effectiveness and humaneness of euthanasia techniques vary considerably across taxa and life stages. Substantial further research is required to validate and optimize humane end-of-life procedures for diverse invertebrate species.

## Simple Summary

Euthanasia is an inevitability in the veterinary profession. The method of euthanasia used must rapidly induce loss of consciousness and death, be effective, and minimize the pain, fear, and distress experienced by the animal. Interference with euthanasia objectives, e.g., research, should be avoided if possible. Moreover, the method of euthanasia should be simple enough to be performed effectively by competent individuals. The availability of peer-reviewed literature specific to invertebrates is scattered and limited. Based on the available scientific evidence, the most effective methods of invertebrate euthanasia entail a two-step approach. Here, the first step consists of the induction of anesthesia to achieve loss of responsiveness, followed by a terminal second step, involving physical or chemical destruction of the brain or major ganglia. The effectiveness and perceived humaneness of euthanasia techniques vary considerably across taxa and life stages.

## 1. Introduction

Invertebrates constitute a paraphyletic grouping of all animals other than those in the chordate subphylum Vertebrata (vertebrates) and are characterized by the absence of a vertebral column. Invertebrates are found in nearly all the world’s habitats, including freshwater aquatic, brackish, marine, and terrestrial environments [1]. They make up nearly 95% of the Earth’s animal species and comprise more than 1.25 million documented species [1,2].

While invertebrates are commonly exhibited in zoological institutions, caught or farmed for consumption, studied in research laboratories, and even kept as pets, evidence-based guidelines for their euthanasia are lacking, despite increasing public and scientific awareness and understanding of invertebrate nociception, perception, and welfare [3,4,5].

The term euthanasia is derived from the Greek words “eu”, meaning “good”, and “Thanatos”, meaning “death”. In this review, methods of “good death”, or euthanasia, are viewed as those methods of terminating life that minimize pain, distress, and anxiety experienced by the animal prior to loss of consciousness, which should be rapidly followed by irreversible cardiac and respiratory arrest and death [2,6,7,8,9,10].

Although the perception of pain requires a conscious experience, defining consciousness, and therefore the ability to perceive pain, across invertebrate species is difficult and the subject of debate [11]. Our imperfect understanding of invertebrate consciousness and nociception should not withhold us from applying high welfare standards for the time being. The history of pain recognition in medicine cautions against assuming the absence of pain perception in the absence of evidence. For example, in human medicine, pain in infants was not widely acknowledged until the 1980s, when long-held Darwinian views of children as less evolved beings and mechanistic interpretations of their pain responses were finally challenged [12]. Similar conceptual biases have delayed the acceptance of pain perception in animals. Over the past decades, veterinary medicine has progressed substantially in this regard: pain is now well-recognized and managed in mammals, and even taxa once thought incapable of experiencing pain, e.g., fish, amphibians, and reptiles, are increasingly acknowledged to possess the neuroanatomical and physiological prerequisites necessary for nociception and pain perception [13,14]. Although distinguishing between a conscious experience of pain and a reflexive nociceptive response remains challenging, best practice dictates presuming the capacity for pain until proven otherwise.

Comparable nociceptive mechanisms have been described in many invertebrate species. While no invertebrates have yet been conclusively demonstrated to experience pain in the vertebrate sense, several possess neural architectures plausibly capable of assigning affective value to noxious stimuli [3,15]. As such, and in light of the lessons learned from the underrecognition of pain in other taxa, it is scientifically and ethically prudent to extend high welfare standards to invertebrates.

This increasing recognition of the potential for pain, suffering, and distress in invertebrates is beginning to be reflected in legislation and policy, although only for select taxa. Notably, Directive 2010/63/EU extends protection to cephalopods used for scientific purposes, based on their presumed capacity to experience pain, suffering, and distress. Similarly, the United Kingdom’s Animal Welfare (Sentience) Act 2022 recognizes cephalopods and decapod crustaceans as sentient animals at the level of central government policy consideration. However, these protections remain limited in scope and exclude the vast majority of invertebrate species. Many other jurisdictions afford no legal protection to invertebrates; for example, the United States Animal Welfare Act explicitly excludes all invertebrate taxa. Consequently, responsibility for ensuring the humane treatment and euthanasia of invertebrates frequently rests on professional guidelines and individual practitioners rather than enforceable legislation, highlighting the need for evidence-based, welfare-oriented recommendations. Therefore, a critical review of the euthanasia methods currently applied to these taxa is long overdue [2,6,9,10,16,17,18].

The American Veterinary Medical Association (AVMA) provides one of the most comprehensive reference documents on methods of euthanasia in invertebrates [6]. Unfortunately, a large proportion of reported methods have been evaluated for effectiveness but not for minimization of stress and adverse reactions. In addition, peer-reviewed evidence supporting these methods are often limited, and many taxa remain unrepresented [6,7,17,19]. The substantial variability in applied methods likely reflects both the ecological and physiological diversity of invertebrates and their various life stages, and the current gaps in our understanding of their biology, evolution, and social organization [16,20].

Writing an impactful review about the euthanasia of all invertebrates would be impossible, as there are approximately 1.25 million documented invertebrate species [1]. Thus, herein we include only information on the metazoan taxa most frequently encountered by veterinarians and researchers [2]. In addition, the scope of this review is limited to techniques intended for the euthanasia of invertebrates. Depopulation methods are therefore largely excluded, as they differ substantially in scale, objectives, and involved welfare considerations.

## 2. Materials and Methods

A search was conducted for publications in academic literature databases, such as PubMed, Scopus, and Web of Science, using subtopic-specific word combinations including “killing”, “euthanasia”, and “invertebrate”, to identify relevant publications. We excluded publications for which full manuscripts were not available, which were not written in English, or which lacked sufficiently detailed information regarding the applied euthanasia method. Subsequently, we critically evaluated reports that we considered relevant for the taxa to be included. The selection and inclusion of publications were guided by the authors’ expert judgment to identify topics aligned with euthanasia methods used in invertebrates. To provide a more comprehensive perspective and highlight connections not always explicitly stated in peer-reviewed literature, grey literature was incorporated in cases where peer-reviewed references were unavailable. As this is a narrative review, data extraction and charting were not carried out, nor was a formal critical appraisal of each source conducted, since these steps were beyond the scope of this manuscript. Limitations of this approach include that some relevant literature may have been missed, and that the search strategy is not perfectly replicable. All provided personal communications by the authors were consistent with standard invertebrate protocols and veterinary practices. Therefore, these handling and sampling procedures were not under the prevue of an Institutional Animal Care and Use Committee (IACUC).

## 3. Considerations in Invertebrate Euthanasia

### 3.1. Humane Euthanasia

The AVMA guidelines for the euthanasia of invertebrates are widely regarded as the authoritative documents on this topic [6]. Within these guidelines, methods of euthanasia are recognized as ‘acceptable’, ‘acceptable with conditions’, or ‘unacceptable’. However, most described methods primarily emphasize procedural effectiveness, with limited consideration of the animal’s subjective experience during the process. In addition, the euthanasia should be carried out in such a way that the environment is not harmed, as there is no sense in euthanasia if the aftermath is harmful to the environment or even the prosecutor. Consequently, several methods deemed acceptable under the AVMA framework may not fully align with the principles of humane euthanasia as defined in this review. In this review, we defined methods of humane euthanasia as those that met all the following criteria: (1) effectiveness, meaning the technique reliably causes death in all target animals; (2) rapid loss of consciousness and/or induction of death; and (3) minimization of perceived pain and distress. Animals may express pain in ways similar to humans, including through avoidance or aggressive behaviors, abnormal postures, guarding to protect an affected area, vocalizations, and physiological and endocrine responses. However, the repertoire of responses to noxious stimuli varies significantly between taxonomic groups [21].

Naturally, euthanasia should always be performed by trained and competent personnel. Although not a determinant of technique humaneness, compatibility with intended animal use and purpose should be considered when choosing a euthanasia method. In most diagnostic and research settings, the preservation of tissue integrity for subsequent analysis is paramount [22]. Moreover, it is important to recognize that complex or resource-intensive protocols may be impractical for those working in low-resource settings. Accordingly, recommended guidelines should remain feasible and implementable even where financial or technical resources are limited.

### 3.2. Considerations for Single-Step Euthanasia Methods

#### 3.2.1. Thermal Methods

Thermal extremes, i.e., boiling, gradual heating, chilling, or freezing, are frequently used in laboratory, aquaculture, and field contexts. Although these methods reliably result in death, they do not induce rapid insensibility, and are therefore considered inhumane [3,9,10,17,23,24,25,26,27,28,29,30]. Chilling and freezing cause a gradual loss of neuromuscular activity rather than immediate insensibility. Moreover, they often elicit prolonged tonic extension in decapods and strong whole-body retraction in gastropods, cues that insensibility is not immediate [28]. This supports classifying thermal methods as inhumane. Primary euthanasia by freezing will also make the determination of death extremely difficult, as some invertebrates can withstand low temperatures by entering torpid or static states that are difficult to distinguish from death. Additionally, many vertebrate animals show nociceptive responses to extreme cold, and homologous ion channels that transduce noxious cold are also found in some invertebrates, suggesting that cold may also be noxious for invertebrates [3]. Euthanasia should not induce prolonged activation of nociceptive pathways. Therefore, temperature-based approaches do not meet our criteria for humane euthanasia. Beyond these welfare concerns, extreme temperatures may compromise tissue integrity, limiting their suitability for histological and molecular analyses. Further research is needed to determine whether death induced by extreme temperatures is inherently noxious to invertebrates.

#### 3.2.2. Chemical Methods

A range of chemical agents are described for the euthanasia of invertebrates. Examples include various herbicides and pesticides, diesel or petrol, hydrogen cyanide, and direct immersion in fixatives, such as formalin or 95% ethanol [24,31,32,33]. However, these substances generally cause prolonged distress and unpredictable physiological effects prior to death. Even acutely toxic insecticides may cause distress and presumed pain before loss of consciousness, rendering them unacceptable for euthanasia [24,34,35]. The use of molluscicides containing metaldehyde or iron phosphate is likewise considered inhumane, as these compounds act slowly and are typically administered without prior anesthesia [31,32].

#### 3.2.3. Gaseous Methods

Evidence remains insufficient to permit an unbiased assessment of the effects of carbon dioxide (CO_2_) exposure on welfare indicators in invertebrates [6]. Although CO_2_ is widely used for the euthanasia of vertebrates, its welfare implications remain controversial even in rodents, with several authors discouraging or advocating prohibition of its use [36,37,38,39,40]. In fruit flies (*Drosophila melanogaster*), CO_2_ has been demonstrated to result in adverse physiological and behavioral effects [41]. In light of current evidence, and in the absence of validation against neural and behavioral endpoints, this method should not be assumed to be humane in invertebrates.

#### 3.2.4. Physical and Mechanical Methods

Physical methods of euthanasia optimally result in direct physical destruction of the brain or major ganglia. Described physical methods include splitting, spiking, vivisection, piercing of ganglia, immersion in fresh- or saltwater baths, soapy water immersion, and drowning. Most of these techniques typically cause a delayed onset of death, prolonged distress, or yield inconsistent results [17,24,25,26,27,28,29,30]. Consequently, their use in non-anesthetized animals should be considered inhumane.

### 3.3. Two-Step Euthanasia

As clearly outlined in the AVMA and CCAC guidelines, a two-step euthanasia process with anesthesia as the first step is preferred whenever possible [3,6,42,43,44]. Acceptable methods of euthanasia render the animal unconscious and insensitive to pain and other adverse effects; however, there must also be assurance of the subsequent death of the animal. Only when there is assurance that circulatory, respiratory, and neurological activity have ceased, should the animal be considered dead.

Recent recommendations by the AZA Aquaitic Invertebrate TAG (2024) propose a two-step euthanasia approach for aquatic invertebrates. Moreover, used agents and concentrations, exposure time, and times to defined endpoints (e.g., righting/withdrawal), should be recorded and shared, aligning clinical practice with research standards [42].

### 3.4. General Considerations When Choosing a Method

Drug regulatory considerations and regional availability should be considered in all cases of chemical euthanasia. In addition, regulatory requirements for the disposal of euthanasia agents, including medicated water, should be taken into consideration.

Where injectable agents are unavailable, the use of inhaled anesthetic overdose has been described as a conditionally acceptable euthanasia method for terrestrial invertebrates [6]. However, supporting references for this recommendation are not provided. Although anesthetic overdose does not require prior anesthesia, the use of a secondary method to ensure or verify death is strongly encouraged. Practical considerations may differ between individual and colony-level euthanasia. For example, groups of aquatic invertebrates can often be anesthetized and kept within the same tank, but whole beehives may not be effectively anesthetized prior to euthanasia.

Depending on the species and facility setup, animals should ideally be euthanized out of sight of conspecifics, or in a separate room. Care must be taken to prevent the transmission of alarm cues, e.g., blood, chemical, auditory or olfactory signals, through shared air or water systems. Regardless of the chosen method, the potential impacts of factors such as body weight, age, sex, season and water temperature on the efficacy of the method must be considered.

A major challenge in invertebrate euthanasia is the reliable verification of death. In vertebrates, confirmation of death typically relies on physiological monitoring techniques, e.g., auscultation, electrocardiography, Doppler ultrasound, or electroencephalography. These methods are often impractical or inapplicable to invertebrates due to fundamental anatomical and physiological differences. Consequently, verification of death commonly relies on behavioral and reflex-based indicators, including the absence of movement, loss of response to external stimuli, lack of resistance to handling, and absence of eye and mouth responses to palpation [6,28].

The choice of secondary (confirmatory) methods should be guided by the purpose of euthanasia and the intended use of postmortem material. In research contexts, the need to preserve tissue integrity for further analysis must sometimes be balanced against welfare considerations [45]. In addition, if chemical methods are elected, appropriate dosing is essential to retain tissue value for further analysis [46]. In cases where animals are intended for human or animal consumption, euthanasia methods must not compromise food safety or render the carcass unsuitable for consumption. Moreover, chemical agents should be avoided when there is a risk of environmental contamination, as pharmaceuticals introduced into waste streams can persist and ultimately enter broader ecosystems, including sources of drinking water.

## 4. Euthanasia Method per Taxa

### 4.1. Coelenterates

Doerr & Stoskopf investigated the euthanasia of moon jellyfish (*Aurelia aurita*) using magnesium salt (MgCl_2_) bath immersion. Immersion in diluted MgCl_2_ at a dose of 142 g/L resulted in death within 32 s of application [47]. Death was defined as the cessation of autonomous bell pulsing and response to external stimuli. The use of MgCl_2_ immersion has been reinforced by other authors [48]. Immersion with other salts, i.e., either potassium chloride (KCl) or magnesium sulfate (MgSO_4_), has been associated with the expulsion of oral mucus, which is considered a sign of acute distress [47]. Although the relatively delayed onset of death observed with MgCl_2_ immersion raises questions regarding its humaneness, no overt stress-associated behaviors were recorded during the study with the described concentrations. In the absence of alternative peer-reviewed data, this method currently represents the most practical option available for the euthanasia of moon jellyfish. Nevertheless, further research should aim to identify and validate methods that achieve a more rapid induction of death while maintaining high welfare standards.

A chemical immobilization method was used to euthanize jellyfish for histologic examination by Freeman et al. [49]. *A. aurita*, *Chrysaora fuscescens*, *C. quinquecirrha*, and *Phacellophora camtschatica* were exposed to a 10% stock solution of eugenol in 95% ethanol administered at a dose of 120 mg/L of water [49]. Jellyfish were exposed to eugenol until they were unresponsive for at least 15 min, with additional drug added as needed. More details were not reported, precluding an evaluation of this method’s effectiveness and humaneness.

### 4.2. Mollusks

Where cephalopods are involved, protocol design should follow FELASA/CephRes/Boyd guidelines on anesthesia and humane euthanasia [8].

Surgical decerebration, i.e., destruction of the brain mass, was performed in stumpy-spined cuttlefish (*Sepia bandensis*) and octopi (*Abdopus aculeatus*, *Octopus bocki*) anesthetized with immersion in 330 mM MgCl_2_ [50]. This technique involves making an incision in the skin between the eyes, exposing the cranium, and then making multiple cuts through the central brain mass with a scalpel. Octopi took approximately 5 min until complete respiratory arrest, which was often preceded by 1–2 min of extremely slow respiration of around 3–4 breaths per minute. Five minutes after breathing had ceased, and an average of 7 min after neural signaling had ceased, decerebration was performed while monitoring signal on the pallial nerve. No behavioral, reflexive or neural responses to skin incision or to surgical destruction of the brain were recorded. A review article on cephalopod mollusks judged immediate mechanical destruction of the brain as a feasible option for the second step [8]. When carried out by highly skilled operators, death by mechanical destruction of the brain is quick, but the nature and degree of any suffering is unknown [51].

An overdose of a non-aversive or non-irritant anesthetic currently represents the most humane non-mechanical method available [6,51]. For Octopoda, a suitable protocol has been described involving immersion in a MgCl_2_ solution for a minimum of 15 min. The MgCl_2_ concentration should be gradually increased to a minimum concentration of 3.5%. The process may be optimized by supplementing with eugenol, the active compound in clove oil, followed by immediate mechanical destruction of the brain to ensure death [51]. These recommendations represent preliminary guidelines that require further refinement, including the development of species-specific protocols [51]. Moreover, susceptibility to magnesium salts may vary among species, underscoring the need for additional validation and standardization of such methods [6].

Benzocaine as an agent of euthanasia has been suggested in the giant Pacific octopus (*Enteroctopus dofleini*) [52]. Concentrations above 2500 mg/L caused terminal anesthesia within 45 min, with concentrations above 3500 mg/L inducing brainstem collapse within 15 min. Although this method proved to be effective, we consider this method to be insufficiently fast to be considered humane.

European succineid snails (*Succinea putris*) were effectively euthanized by a 2-step method and showed to meet all welfare and scientific requirements [53]. This 2-step method included a first induction of anesthesia by immersion in 5% ethanol, followed by immersion in a euthanizing and tissue-preserving solution of 70% to 95% ethanol or 10% neutral buffered formalin. Alternative chemical methods of euthanasia for terrestrial snails commonly used in field research, e.g., non-anesthetized immersion in concentrated ethanol or formalin, are considered to be unacceptable due to the presumed noxious effect of these solutions to non-anesthetized animals.

### 4.3. Crustaceans

The best practice for euthanizing decapod crustaceans has been debated for a century [28,54,55]. Recent research indicates a shift away from live boiling toward more humane techniques such as electrical stunning and mechanical destruction of the central nervous system [28,55,56].

Electrical stunning was demonstrated to be a feasible a second step technique to euthanize Norway lobsters (*Nephrops norvegicus*), brown crabs (*Cancer pagarus*), New Zealand rock lobsters (*Jasus edwardsii*), and freshwater crayfish (*Paranephrops zealandicus*) [57,58,59]. The effectiveness of electrical stunning depends on the applied electrical parameters, i.e., waveform, minimum current and voltage, maximum frequency, and minimum time of exposure. These parameters need to be adjusted to the species, size, developmental stage, and stage of molt of the animal. Larger crustaceans with thick exoskeletons require higher voltages than smaller species, such as prawns and shrimp [55]. Electrical stunning has been demonstrated to cause immediate death in a proportion of individuals, while effectively abolishing central nervous activity, sensory responsiveness, and motor function in others [57,58,59]. Moreover, no differences in L-lactate levels were reported between stunned and control animals in one study, suggesting no measurable stress occurred with stunning [28]. Standardized electrical dosage regimens that reliably induce instantaneous death across species and sizes have yet to be established. Until such parameters are validated, electrical stunning should not be considered a stand-alone euthanasia method, and a secondary step to ensure death is recommended [55].

Mechanical destruction of the central nervous system by splitting or spiking is also widely used and considered effective when performed by trained personnel [17,28]. Splitting involves rapidly making a longitudinal incision along the midline of the head and thorax with a sharp blade to sever the main chain of ganglia. Similarly, spiking is used in crabs, which have a more centralized nervous system consisting of two anterior ganglia. Both ganglia must be swiftly pierced and destroyed using an awl. In both cases, effective application depends on the skill of the operator to ensure complete destruction of neural tissue, thereby minimizing suffering [17,28]. Although both mechanical methods are considered fast and effective when performed by skilled personnel, no studies were identified that evaluated animal responsiveness or welfare using the described techniques. As a result, it cannot be guaranteed that neural activity ceases immediately, and these techniques should only be utilized as a second step in two-step euthanasia.

Clove oil immersion is reportedly effective in Australian giant crabs (*Pseudocarcinus gigas*), with no reported signs of distress [27]. The injection of eugenol has similarly been described to effectively euthanize American lobsters (*Homarus americanus*) and green crabs (*Carcinus maenas*) at a dose of 7 μL/gm for lobsters and 10 μL/gm for crabs [60]. No behavioral indicators of distress were observed with the intrapericardial injection of eugenol in lobsters or crabs. Moreover, it has been described that seasonality may affect the required dosage for effective euthanasia in some species [60]. High-dose eugenol injection is presently considered a humane euthanasia method based on available evidence, although additional neurophysiological studies are needed to confirm the rapid cessation of neural activity.

**Figure 1:**
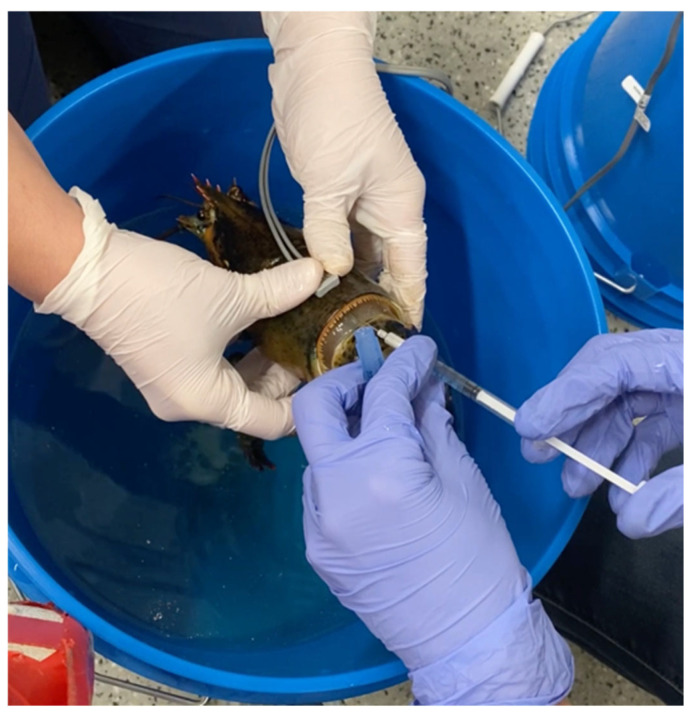
Photo of an American lobster being euthanized (second step) with intracardiac KCl 10 mEq/kg after immersion anesthesia with eugenol. No behavioral indicators of distress were observed (photograph provided by G.A. Lewbart).

In American lobsters (*Homarus americanus*), the injection of 1000 mg/kg KCl into the base of the second walking legs, targeting the hemolymph sinus surrounding the ventral nerve cord, was investigated as a euthanasia method. Death was defined as time of cardiac arrest, as viewed and measured by use of ultrasound. This method was demonstrated to be effective and reliable. However, cardiac arrest only followed within 60 to 90 s after injection. Consequently, this method does not fit our criteria of humane euthanasia, as it is not rapid. Moreover, histological lesions were observed post-mortem due the chemical method [61]. American lobster can be euthanized with intracardiac KCl (10 mEq/kg) after anesthesia with immersion eugenol (Lewbart, pers. comm., Figure 1).

For smaller aquatic crustaceans, such as brine shrimp (*Artemia franciscana*), immersion in euthanasia solutions of 70–95% alcohol or 10% neutral buffered formalin have been tested as part of a two-step euthanasia process [62]. While these methods were effective in inducing death, all treatments were associated with abnormal behaviors prior to death, suggesting distress. Consequently, these methods cannot currently be considered humane, and no effective or humane euthanasia techniques are described for small aquatic crustaceans at present.

### 4.4. Echinoderms

**Figure 2:**
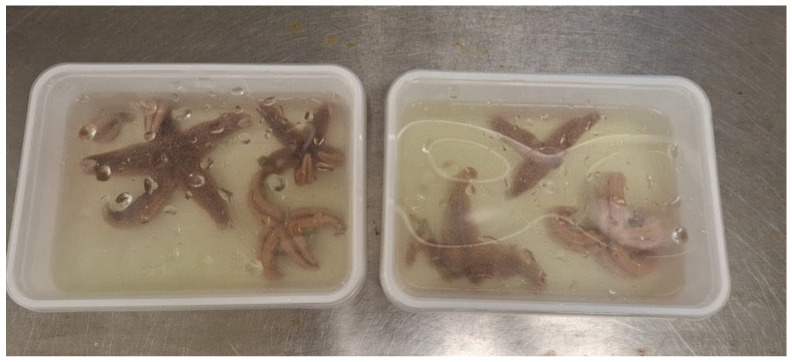
Example of common sea stars (*A. rubens*) euthanized by extended immersion in eugenol (photograph provided by R.A. Nederlof).

An effective two-step euthanasia technique has been reported for adult common sea stars (*Asterias rubens*), in which the animals were first immersed in a 75 g/L MgCl_2_ solution. Sea stars were left in the MgCl_2_ solution for 1 min past complete loss of response to tactile stimulation, intending to go beyond the anesthetic effects of MgCl_2_ immersion. Once sea stars were rendered insensible, they were immediately dissected with sharp scissors at the junction of the aboral and oral body walls. No sea stars regained spontaneous movement or response to stimuli during sampling. It took approximately 5 min to completely sample each sea star after removal from the MgCl_2_ solution. In this method, high-quality RNA samples useful for molecular diagnostics could be obtained from the pyloric ceca [63]. Extended immersion (>20 min) in eugenol or 2-phenoxyethanol (0.8 mg/L) effectively euthanizes common sea stars, though death may be delayed with lower dosages, and a physical secondary step of euthanasia is recommended to ensure death (Nederlof, pers. comm. Figure 2). While these immersion techniques are effective and associated with anesthetic properties, the delayed onset of death and potential for residual responsiveness make them less desirable as sole euthanasia methods. Other described acceptable secondary methods for sea stars include immersion in 70% alcohol, immersion in 10% formalin or pithing [6]. The secondary step can be altered depending on the ultimate goal for sample analyses. For example, if histology is desired, placement in 10% formalin as a second step would facilitate both killing the sea star and sample fixation. However, formalin results in crosslinks that chemically modify RNA and preclude isolation and evaluation of RNA. When possible, the second step in a two-step method of euthanasia should result in destruction of the central nervous system. The echinoderm circumoral nerve ring and radial nerve cords are considered the central nervous system. However, there is no evidence that these are coordinating structures, rather the circumoral nerve ring around the mouth appears to provide connections between radial nerve cords that extend down the arms of sea stars [63]. Common sea stars do not have nervous tissue that is visible with the naked eye, which precludes the ability to pith them. However, the sampling method performed by Wahltinez et al. presumptively resulted in the rapid destruction of the majority of nervous tissue [63].

In chocolate chip sea cucumbers (*Isostichopus badionotus*), evisceration has been described, complying with the Mexican Official Standard for the Care and Use of Laboratory Animals and with the rules of the Internal Committee for the Care and Use of Laboratory Animals [64]. However, in the absence of data evaluating this technique’s impact on animal responsiveness or neural activity, it should not be performed without prior anesthesia or desensitization [42].

There is limited literature on the chemical euthanasia of echinoderms. The use of sodium pentobarbital has been reported in black sea cucumbers (*Holothuria atra*), though methodological and behavioral responsive details were not provided [65].

### 4.5. Chelicerates

#### 4.5.1. Aquatic Chelicerates

In Atlantic horseshoe crabs (*Limulus polyphemus*), mechanical destruction of the dorsal ganglion located on the dorsal midline between the eyes has been described [66]. The success of this method is highly dependent on proper user technique, and time to cessation of neurological activity and death after crushing are unknown.

Published recommendations for euthanasia specific to Atlantic horseshoe crabs include pentobarbital injection (390 mg/mL, 1–2 mL/animal) directly into the cardiac sinus via the arthrodial membrane [66,67]. The intracardiac administration of eugenol at 2–4 mL/kg has also been described as a relatively fast and effective means of euthanasia in this species [68]. However, the time to loss of responsiveness, righting reflex, and spontaneous movement typically ranged from 2 to 5 min, and higher doses may be required in some individuals. Due to this delayed onset and potential variability in response, intracardiac eugenol injection should not be considered a humane single-step euthanasia method, and prior anesthesia is recommended.

#### 4.5.2. Terrestrial Chelicerates

For terrestrial invertebrates, an inhalant agent should be used for inducing anesthesia. Once the spider is anesthetized, pentobarbital can be injected into the haemocoel [6,9,23,69]. Ideally, pentobarbital is injected directly into the circulating hemolymph. However, due to the open circulatory systems of most invertebrates, true intravascular administration is often impractical or impossible; in such cases, intracoelomic injection is recommended.

For spiders, general anesthesia followed by immersion in 70% ethanol is also commonly applied, and allows for superior tissue preservation [23,69].

In another study, euthanasia was achieved after immobilization through the use of an injection of KCl, causing death through terminal depolarization of the thoracic ganglia because of hyperkalosis [70]. For euthanasia of theraphosid spiders, either a dose of 0.5% *v*/*w* 300 mg/mL KCl can be administered centrally via the sternum into the prosomal ganglia or 1% *v*/*w* 300 mg/mL KCl can be delivered intracardiac. This method is effective in ablating the nervous system and is non-recoverable [69,70].

Barbiturate injection into the pericardial sinus of spiders under general anesthesia has been described [23]. To this end, the animal is placed in ventral recumbency, and a needle is introduced at a 45-degree angle into the dorsal midline of the cranial third of the opisthosoma. An alternate euthanasia method used was prosomal injection into the sub-esophageal ganglion with the spider in dorsal recumbency [23]. A needle can be passed through the ventral prosomal cuticle until the endostermite is hit; the needle can be drawn back slightly to the site of the ganglion where either KCl or barbiturates can be administered [70]. This method may be preferable to the intracardiac method in dehydrated or debilitated theraphosids. A dose of >400 mg/kg of sodium pentobarbital with either method has been described [23]. Both methods are described to be quick and reliable, as the heart stops within seconds, as determined through ultrasonography. Presently, this constitutes the technique most closely aligning with our definition of humane euthanasia, so long as the animal is anesthetized first.

Some hobbyist literature has recommended rapid freezing for euthanasia, but this will compromise tissues for histopathological examination and the formation of ice crystals in inappropriately anesthetized animals may be perceived as a noxious stimulus [9,10,23,69,71,72].

Death can be confirmed with a Doppler probe demonstrating permanent cessation of circulation and heart rate. Alternatively leaving the spider for 24 h on a drawn outline can add further confidence that the spider is dead in the absence of a Doppler probe [23].

In scorpions (*Mesobuthus gibbosus*), lethal exposure to chloroform or diethyl ether has been anecdotally reported as a euthanasia method [73]. However, these reports lack details regarding time to death, behavioral responses, or cessation of neurological activity. Consequently, it remains unclear whether such methods meet the criteria for humane euthanasia.

### 4.6. Myriapods

Most methods described in the literature for the euthanasia of myriapods are considered inhumane, e.g., submersion in 95% ethanol for 10 min without prior anesthesia [74]. Consequently, recommendations for the euthanasia of myriapods are consistent with those for terrestrial chelicerates, although peer-reviewed literature is lacking [6].

### 4.7. Insects

Several studies have focused on the euthanasia of bees, considering practicality, user safety, and environmental impact [24,34,35]. A 2020 survey identified ten commonly applied methods [24]. Out of those ten, nine are not considered to be humane by our standards due to the welfare concerns mentioned with the use of these techniques in other invertebrate taxa. The non-humane methods were diesel poisoning, petrol poisoning, hydrogen cyanide poisoning, long-term exposure to negative cold (“freezing”); suffocation of the swarm exposed to the sun; exposure to cold and CO_2_ (“dry ice”); suffocation by topical surfactant solution (“soapy water”); intoxication by topical alcohol solution; and suffocation by sustained immersion in water (“drowning”).

We identified the use of sulfur dioxide (SO_2_) as the most humane method to euthanize bees in a hive [24]. When employing this technique, operators must use appropriate personal protective equipment and ensure that the hive is fully gas-tight; moreover, euthanasia of brood requires an additional secondary step. Euthanasia of the whole adult colony is described to be achieved after 15 min of SO_2_ exposure. However, as is often the case in invertebrate research, this research solely focused on the effectiveness of the applied method and did not evaluate animal behavioral or physiological responses to SO_2_ exposure. Though effective, we cannot confirm that this method aligns with our criteria for humane euthanasia.

For cockroaches, current recommendations include freezing, immersion in alcohol or formaldehyde, or injection with drugs such as pentobarbital or potassium chloride [6]. A study conducted in four species of cockroaches, dubia (*Blaptica dubia*), red runner (*Shelfordella lateralis*), Madagascar hissing (*Gromphadorhina portentosa*), and giant cave (*Blaberus giganteus*) cockroaches evaluated the following methods: freezing at −18 °C or −80 °C from 0.25 to 24 h; immersion in 10% neutral buffered formalin, 70% isopropyl alcohol, or reverse osmosis water for 0.25 or 0.5 h; or intracoelomic injection of KCl (456 mEq/kg) or pentobarbital-based euthanasia solution (3.9 mg/kg) [75]. Following all treatments, cockroaches were monitored for an additional 24 h for spontaneous movement. Irreversible loss of movement was considered synonymous with death. In this study, only isoflurane anesthesia followed by either 70% isopropyl alcohol immersion for 0.25–0.5 h or isoflurane exposure for 24 h resulted in the euthanasia of all animals. Although no information is available on physiological or neurological responses to these methods, we recommend 70% isopropyl alcohol immersion as the second euthanasia step in cockroaches based on reported effectiveness, swiftness, and lack of visible response. Future research, however, should aim to verify the absence of neurological or hormonal signals that may be associated with a stress response.

Another study evaluated soapy water and freezing as humane euthanasia methods, and both methods were found to be consistently effective in cave and Madagascar hissing cockroaches [76]. These methods, however, do not align with our criteria for humane euthanasia due to the concerns described in Section 3.2.1 and Section 3.2.4. Moreover, sporadic failures occurred, necessitating an additional euthanasia step.

The injection of ivermectin (100 mg/kg) is reported to be effective for the euthanasia of isoflurane-anesthetized male and female thorny devil stick insects (*Eurycantha calcarata*) [77]. For administration, insects were placed in dorsal recumbency, and a 25 g needle attached to a 1 mL syringe containing the ivermectin was inserted with the bevel facing upwards along the ventral midline between the first leg plate and the caudal adjacent ventral plate. Death was defined as the absence of spontaneous movement for 48 h. In the same study, an injection with KCl (200 mEq/kg) was demonstrated to be unreliable [77].

Lum & Keller investigated the effectivity of several euthanasia methods of darkling beetle larvae (*Zophobas atratus*) [78]. First, larvae were successfully anesthetized using isoflurane. As the second-step, only the injection of KCl at a dose of 3 mg/g body weight and immersion in pure ethanol resulted in 100% mortality. The injection was performed intracoelomic on the ventral midline between the second and third segments using a U-100 insulin syringe but resulted in spontaneous movement of the animals, suggesting a reaction to the noxious stimulus. This was not observed in animals submerged in pure ethanol. Subjects that did not move at 96 h were considered deceased [78].

As with terrestrial chelicerates and myriapods, other recommended methods for insects include pentobarbital injection, or exposure to high concentrations of CO_2_ or volatile anesthetic agents [6]. However, no peer-reviewed studies have yet validated the efficacy or humaneness of these approaches in insects.

**Figure 3:**
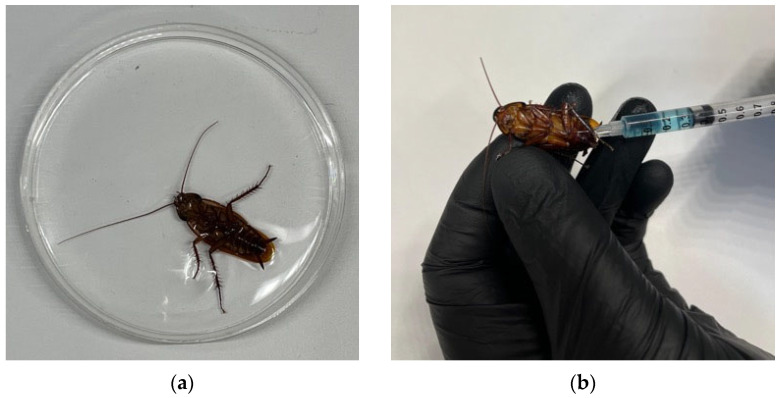
Euthanasia of American cockroach (*Periplaneta americana*) using (**a**) 70% alcohol immersion, and (**b**) injection with 0.30 mL pentobarbital (Euthasol^®^, 500 mg/mL). In alcohol, death occurred within seconds, without behavioral signs of distress. Following pentobarbital injection, most animals remained alive until immersed in alcohol after 60 s of behavioral response (photographs provided by J.B.G. Stumpel).

American cockroaches (*Periplaneta americana*) were euthanized by immersion in 70% alcohol or by injection of pentobarbital (0.30 mL of pentobarbital, 500 mg/mL). In alcohol, death occurred within seconds, and no distress was observed. However, following pentobarbital injection, most animals remained alive, demonstrating behavioral signs of distress until immersion in alcohol after 60 s (Stumpel, pers. comm., Figure 3a,b).

Based on the current literature, it remains difficult to provide definitive euthanasia recommendations applicable across all insect taxa, and substantial additional research is needed. In the absence of species-specific data, the cockroach protocol using 70% ethanol immersion as the secondary euthanasia step also appears to be the most broadly applicable approach for other insect species.

### 4.8. Annelids

Annelids (segmented worms) are widely used as experimental models in ecotoxicology, neurobiology, and regenerative biology. Nevertheless, published guidance on humane anesthesia and euthanasia for these invertebrates remains limited.

In polychaetes, or bristle worms, anesthesia has been reported using 7–8% MgCl_2_, MS-222, phenoxyethanol, clove oil, or 10% carvacrol [79,80,81,82]. Euthanasia of polychaetes has most commonly been performed by immersion in 4–5% formaldehyde for 24 h [79,80].

In oligochaetes, or aquatic and terrestrial worms, anesthesia has been described using 5% ethanol [83]. Euthanasia of earthworms (*Eisenia fetida* and *Dendrobaena veneta*) has been achieved with 4% solutions of procaine or lidocaine. However, pronounced interspecific differences in median lethal concentration (LC_50_) were observed [84]. Moreover, exposure to these solutions elicited sudden movements and the expulsion of coelomic fluids, suggesting nociceptive responses [84]. Therefore, if this method is elected, prior anesthesia should be pursued to safeguard animal welfare.

In hirudineans, or leeches, anesthesia has been reported using 400 mg/L benzocaine or saturated mephenesin solutions [85,86]. Subsequent euthanasia has been described through immersion in 70% ethanol [87,88].

Overall, recommendations for euthanasia across annelid taxa are largely extrapolated from limited and heterogeneous literature, and objective evaluations of animal welfare outcomes, effectiveness, and rapidity of death are notably absent for nearly all described techniques. As a result, no definitive conclusions can be drawn regarding the humaneness of the currently reported methods. The primary exception is the documentation of adverse behavioral responses in oligochaetes exposed to procaine and lidocaine solutions, which suggests the potential for nociceptive stimulation and underscores the importance of prior anesthesia when such agents are used [84]. Additionally, although not formally assessed in the literature, complete mechanical destruction of the animal may be considered as an alternative second step when compatible with experimental objectives, provided that it does not affect the euthanasia goal, e.g., research.

## 5. Discussion

Growing awareness of animal welfare, including increasing concern for the welfare of invertebrates, underscores the responsibility of veterinarians, researchers, and animal care professionals to ensure that invertebrates are euthanized using the most humane methods available. Euthanasia should achieve rapid and irreversible loss of consciousness while minimizing or eliminating presumed pain and distress. To this end, we recommend a two-step euthanasia procedure in most circumstances (Table 1). The first step involves the induction of anesthesia to achieve loss of responsiveness, followed by a terminal method involving physical or chemical destruction of the brain or major ganglia. This review highlights the need for research investigating the relative welfare impacts of handling, anesthesia, and euthanasia procedures, as the current scarcity of data precludes any definitive conclusions on these aspects.

This review highlights the diversity of methods currently reported across invertebrate taxa, as well as significant gaps in the scientific evidence supporting their humaneness and effectiveness. Although different techniques are described, few have been evaluated for humaneness, and many taxa remain without well-supported recommendations. Clear and transparent reporting of the methods used for euthanasia and confirmation of death should therefore become standard practice in scientific publications. Besides evaluating technique effectiveness, future research should aim to incorporate behavioral and neurophysiological analyses to evaluate the potential adverse welfare effects.

Based on the existing literature, an overview of recommended euthanasia techniques is provided in Table 1. However, these recommendations should be regarded as provisional rather than definitive standards. As discussed in the preceding sections, scientific evidence supporting the humaneness of euthanasia methods remains limited or absent for most invertebrate phyla, and the guidance presented here is therefore subject to revision as additional data become available. In practice, the most appropriate euthanasia technique may differ from those summarized in Table 1 depending on contextual and biological factors, including whether procedures are applied to individuals or groups, the potential for transmission of alarm cues between conspecifics, and species-specific physiological characteristics. Further considerations include intrinsic biological variables such as body mass, age, sex, developmental stage, metabolic rate, season, and ambient or water temperature, as well as extrinsic factors such as the intended purpose of euthanasia and the risk of environmental contamination. Collectively, these factors highlight the need for the cautious interpretation and application of existing protocols and underscore the importance of tailoring euthanasia methods to the specific species, setting, and objectives involved.

There is a growing need, but a clear lack of evidence-based resources, for the establishment of species-specific, scientifically validated guidelines for the humane euthanasia of invertebrates. ARRIVE (Animal Research: reporting of in vivo experiments; details can be found on https://arriveguidelines.org/arrive-guidelines, accessed on 8 January 2026) guidelines established by the National Center for the replacement, refinement, and reduction of animal research (NC3Rs, London, UK) have a series of recommendations that encompass experimental design, data analysis, and a description of details to report for experimental procedures [89]. The experimental procedure section of the ARRIVE guidelines covers euthanasia and describes in detail what should be outlined in manuscripts, including: euthanasia method and which standards the methods should comply with, the anesthetic choice and dosage, measures taken to reduce pain and distress, timing of euthanasia, and timing of tissue collection after euthanasia. The goals of the ARRIVE guidelines are to maximize the quality and reliability of published research wile maintaining animal welfare standards and to enable others to better scrutinize, evaluate, and reproduce it.

All personnel performing procedures for euthanasia must be trained and competent to ensure that euthanasia is carried out in the most humane manner. Training should include species-appropriate handling and care procedures to minimize stress and suffering, recognition of species-specific signs of pain and distress using behavioral measures, proper methods of handling and restraining the animal, proper application of the method of euthanasia and use of equipment, recognition and assessment of unconsciousness, and ensuring, and recognition and confirmation of death [43,90].

Further research is essential to advance our understanding of potential pain perception and distress in invertebrates, and to ensure that humane, safe, and environmentally responsible practices are implemented across all settings where invertebrates are euthanized. From a methodological perspective, priority should be given to studies that incorporate behavioral and neurophysiological measures to evaluate welfare outcomes across species and life stages. From a regulatory perspective, efforts should focus on the development of guidelines that establish clear standards for invertebrate welfare across a broader range of taxa. The ongoing challenge of distinguishing pain perception from nociception in these taxa should not be used as justification for lowering welfare standards, and research should continue to adopt a precautionary framework that presumes the capacity for suffering in invertebrates. History reminds us of the ethical pitfalls of viewing species through a purely Darwinian or mechanistic lens. As the body of evidence supporting pain perception across vertebrates continues to grow, prudence dictates that we extend the same ethical caution to invertebrates, at least until scientific evidence indicates otherwise. A precautionary approach that prioritizes animal welfare, even in the absence of conclusive evidence, represents both a scientifically sound and ethically responsible framework for future work in this field.

**Table 1:** Recommended euthanasia techniques for different invertebrate phyla.

Section	Described Species	Advised Euthanasia Method Include Medicine, Dosage, and Route	Two-Step Euthanasia Protocol Recommended?	Reference
3.1 Coelenterates	Jellyfish	Step 1: MgCl_2_ immersion (142 g/L) Step 2: Physical destruction or immersion in fixative	Yes	[47]
	Anemones	Step 1: MgCl_2_ immersion Step 2: Physical destruction or immersion in ethanol or fixative	Yes	[91]
	Corals	Step 1: MgCl_2_ immersion Step 2: Physical destruction or immersion in ethanol or fixative	Yes	[91]
3.2 Mollusks	Gastropods	Step 1: 5% ethanol immersion (10–15 min) Step 2: 70–95% ethanol or 10% formalin immersion	Yes	[53]
	Squids	Step 1: MgCl_2_ immersion Step 2: Mechanical destruction of the brain	Yes	[51] [50]
	Cuttlefish	Step 1: MgCl_2_ immersion Step 2: Surgical decerebration	Yes	[8]
	Octopi	Step 1: MgCl_2_ immersion Step 2: Surgical decerebration	Yes	[8]
3.3 Crustaceans	Lobsters	Eugenol prolonged immersion or injection	No	[60]
	Crabs	Step 1: Eugenol prolonged immersion or injection Step 2: Physical destruction or freezing	Yes	[60]
	Crayfish	Electrical stunning	No	[59].
	Shrimp	No validated humane methods available		[62]
3.4 Echinoderms	Sea stars	Step 1: MgCl_2_ immersion Step 2: Physical destruction	Yes	[63]
	Urchins	Step 1: MgCl_2_ immersion Step 2: Immersion in ethanol or physical destruction		[9]
	Sea cucumbers	Step 1: MgCl_2_ immersion Step 2: Evisceration	Yes	[64]
3.5 Chelicerates	Spiders	Step 1: isoflurane anesthesia Step 2: Immersion in >70% ethanol	Yes	[23]
	Scorpions	Step 1: isoflurane anesthesia Step 2: Immersion in >70% ethanol	Yes	[9]
	Horseshoe crabs	Step 1: Pentobarbital injection (390 mg, 1–2 mL/animal) into the cardiac sinus Step 2: Physical destruction	Yes	[66,67]
3.6 Myriapods	Centipedes and millipedes	Step 1: isoflurane anesthesia Step 2: Immersion in >70% ethanol	Yes	[9]
3.7 Insects	Honey bees (colony)	SO_2_ exposure	No	[24]
	Cockroaches	Step 1: 70% isopropyl alcohol immersion Step 2: Physical destruction	Yes	[75]
	Stick insects	Step 1: isoflurane anesthesia Step 2: ivermectin injection (100 mg/kg)	Yes	[77]
	Beetle larvae	Step 1: isoflurane anesthesia Step 2: immersion in 100% ethanol		[78]
3.8 Annelids	Polychaetes	Step 1: 7–8% MgCl_2_ immersion Step 2: prolonged 4–5% formaldehyde immersion	Yes	[79]
	Oligochaetes	Step 1: 5% ethanol immersion Step 2: immersion in 4% procaine or lidocaine	Yes	[83,84]
	Hirudineans	Step 1: immersion in 400 mg/L benzocaine Step 2: 70% isopropyl alcohol immersion	Yes	[86,88]

## 6. Conclusions

While this review was prepared with a great deal of scrutiny, it is not intended to be an instruction manual. Decisions regarding the best method of euthanasia should consider the competence of the personnel involved and the species, age, and health status of the animal, as well as the local availability of and legislation surrounding the proposed techniques.

We demonstrate that many published scientific papers do not provide an adequate description of the euthanasia methods. Further work needs to be conducted in order to establish whether or not these methods meet the humane euthanasia criteria. Recommendations from our review include the use of a two-step euthanasia method in nearly all instances. Death must be verified following euthanasia and prior to disposal of the animal. Moreover, we urge researchers and veterinary professionals to more elaborately describe the details of used euthanasia techniques, as well as their effects on animal behavioral and physiological parameters.

Based on the available literature, we summarized the euthanasia methods most strictly adhering to our definition of humane euthanasia per species in Table 1. It is important to note that minimal species-specific literature exists, and that even recommended methods may not be supported by thorough behavioral and neurophysiological analyses that validate their humaneness. Consequently, this review emphasizes the need for methodological validation, improved techniques to assess invertebrate welfare during euthanasia, and the expansion of described techniques to novel species.

## Data Availability

Data are available upon reasonable request.
